# Biofilm Attached Cultivation of *Chlorella pyrenoidosa* Is a Developed System for Swine Wastewater Treatment and Lipid Production

**DOI:** 10.3389/fpls.2017.01594

**Published:** 2017-09-21

**Authors:** Pengfei Cheng, Yuanzhu Wang, Tianzhong Liu, Defu Liu

**Affiliations:** ^1^Poyang Lake Eco-economy Research Center, Jiujiang University Jiujiang, China; ^2^School of Water Resources and Hydropower Engineering, Wuhan University Wuhan, China; ^3^School of Water Conservancy and Environment, China Three Gorges University Yichang, China; ^4^Qingdao Institute of Bioenergy and Bioprocess Technology, Chinese Academy of Sciences Qingdao, China; ^5^School of Architectural and Environment, Hubei University of Technology Wuhan, China

**Keywords:** *Chlorella pyrenoidosa*, swine wastewater, biofilm attached cultivation, heavy metals, lipid

## Abstract

This study showed the new potential of using soluble contents and heavy metals in swine wastewater as nutrient supplements for the algae *Chlorella pyrenoidosa* with biofilm attached method. Algae with biofilm attached cultivation grew well in unpasteurized wastewater reaching a biomass productivity of 5.03 g m^−2^ d^−1^, lipid content of 35.9% and lipid productivity of 1.80 g m^−2^ d^−1^. *Chlorella* grew in BG11 medium delivered lower values for each of the aforementioned parameters. The FAMEs compositions in the algae paste were mainly consisted of C16:0, C18:2, and C18:3. Algae removed NH_4_^+^–N, total phosphorus (TP), and COD by 75.9, 68.4, and 74.8%, respectively. Notably, Zn^2+^, Cu^+^, and Fe^2+^ were removed from wastewater with a ratio of 65.71, 53.64, and 58.89%, respectively. Biofilm attached cultivation of *C. pyrenoidosa* in swine wastewater containing heavy metals could accumulate considerable biomass and lipid, and the removal ratio of NH_4_^+^–N, TP, COD, and as well as heavy metal were high. Treatment of wastewater with biofilm attached cultivation showed an increasingly popular for the concentration of microalgae and environmental sustainability.

## Introduction

Large quantities of waste continuously produced from the intensive livestock industries worldwide represent an increasingly concerning threat to the environment, especially when they were not treated properly. Excessive nutrients in the piggery wastewater, such as, nitrogen and phosphorus, will cause eutrophication in natural water bodies (Cai et al., 2013; Ayre et al., 2017). Compared with municipal domestic sewage-based wastewater, swine wastewater, which is often derived from manure, may contain very high amounts of N and P and may also contain toxic metals (Sturm and Lamer, 2011; Zhou et al., 2012). The principle in wastewater treatment is the reduction of nutrient and toxic metal to acceptable limits prior to discharge and reuse (Miranda et al., 2017). The majority of examples for wastewater treatment technologies were based on chemical or physical methods that were not economical for livestock wastewater treatment (Xu et al., 2015; Li et al., 2016).

As we known, microalgae are considered promising for production of biofuels and higher value products, but require a lot of nutrients in their growth, resulting in high operation costs and possibly adverse environmental effects due to nutrient leakage into the environment (Mata et al., 2010; Borowitzka and Moheimani, 2013; Shah et al., 2016). Therefore, application of wastewater rich in nutrients, such as, nitrogen and phosphorus, for fertilizer-driven microalgal cultivation is a promising approach to enhance economic and environmental sustainability (Mulbry et al., 2008). In particular, the genus *Chlorella* is commonly used in the wastewater treatment system due to their high tolerance to soluble organic compounds.

Nitrogen and phosphorus are removed from wastewater in two separate processes in conventional wastewater treatment. Nitrogen is usually converted into N_2_ gas through coupled nitrification–denitrification, whereas phosphorus is precipitated with metal salts. By contrast, nitrogen and phosphorus could be removed from wastewater in a single process with the use of microalgae (Christenson and Sims, 2011; Abdelaziz et al., 2013; Beuckels et al., 2015). Microalgae absorb nitrogen and phosphorus from swine wastewater and convert these nutrients into biomass. Kothari et al. (2012) found that *Chlorella pyrenoidosa* could remove about 80–85% total phosphorus (TP) and 60–80% of total nitrogen (TN) from dairy wastewater. Markou et al. (2012) also found that the maximum removal of chemical oxygen demand (COD) was 73.18%, while phenols, phosphorus, and nitrates in some runs were completely removed when cultivating *Arthrospira platensis* in olive-oil mill wastewater.

However, studies of microalgae-based wastewater treatment were generally based on commercial microalgae cultivation systems, such as, open ponds or closed photobioreactors (PBRs), which exhibited relatively low biomass productivity, high water requirements and high liquid transportation and cumbersome and higher costs in harvesting (Ozkan et al., 2012; Berner et al., 2015). This research, we introduced a novel cultivation system, which was called “biofilm attached cultivation” by Liu et al. (2013). Unlike traditional cultivation methods based on suspended cultures of cells in aqua-medium, the biofilm attached cultivation is a different technology in which the algal cells are immobilized and settled on artificial supporting materials in high density. In brief, microalgal cells are attached to a supporting structure to form an artificial “leaf” and multiple of these leaves are vertically inserted into a glass chamber in this system (Cheng et al., 2013; Liu et al., 2013). Biofilm attached cultivation of microalgae in wastewater separated algae cells from wastewater instead of concentrating or filtering, and the treated wastewater can be recycled immediately. However, there were rarely reports that investigated the feasibility of using wastewater as nutrient supplements for biofilm attached cultivation of microalgae.

In this study, an integrated approach which combined freshwater microalgae *C. pyrenoidosa* biofilm attached cultivation with heavy metals rich swine wastewater treatment was investigated. The objectives of our study were: (1) to determine a feasibility study with undiluted piggery wastewater for algal cultivation, (2) to specify the productivities of biomass, lipids, and FAMEs, and (3) to reveal relevant nutrient and heavy metals removal abilities.

## Materials and methods

### Algal strain and inoculum preparation

The microalgae strain *C. pyrenoidosa* which was acclimated in poultry wastewater, was obtained from Hubei University of Technology. As we mentioned before, in preparing the inoculum for biofilm attached bioreactors, the alga was first cultivated in glass bubbling columns (diameter = 0.05 m) until the exponential growth phase was reached (approximately 5 days). The glass columns contained 0.6 L of algal broth and were continuously illuminated by cold-white fluorescent lamps (NFL28-T5, NVC, China) with a light intensity of 100 ± 10 μmol m^−2^s^−1^. The broth temperature was 20 ± 2°C. CO_2_ enriched air (1% v/v) was continuously injected into the bottom of the columns with a speed of 1 vvm (0.7 L/min for each column) to agitate the algal broth as well as supply carbon (Cheng et al., 2013).

### Culture medium and photobioreactor

In this research, the algae cultivated in glass bubbling columns and biofilm attached cultivation system (control) was grown in a BG11 medium (Chiu et al., 2015), each liter of which contains 1.5 g NaNO_3_, 0.075 g MgSO_4_·7H_2_O, 0.036 g CaCl_2_·2H_2_O, 0.04 g KH_2_PO_4_·H_2_O, 0.02 g Na_2_CO_3_, 6.0 × 10^−3^ g citric acid, 1.0 × 10^−3^ g Na_2_EDTA, 6.0 × 10^−3^ g ferric ammonium citrate, 2.22 × 10^−4^ g ZnSO_4_·7H_2_O, 6.9 × 10^−5^ g CuSO_4_·5H_2_O, 1.81 × 10^−3^ g MnCl_2_·4H_2_O, 3.9 × 10^−4^ g Na_2_MoO_4_·2H_2_O, 4.94 × 10^−5^ g Co(NO_3_)_2_·6H_2_O, and 2.86 × 10^−3^ g H_3_BO_3_.

**Figure 1:**
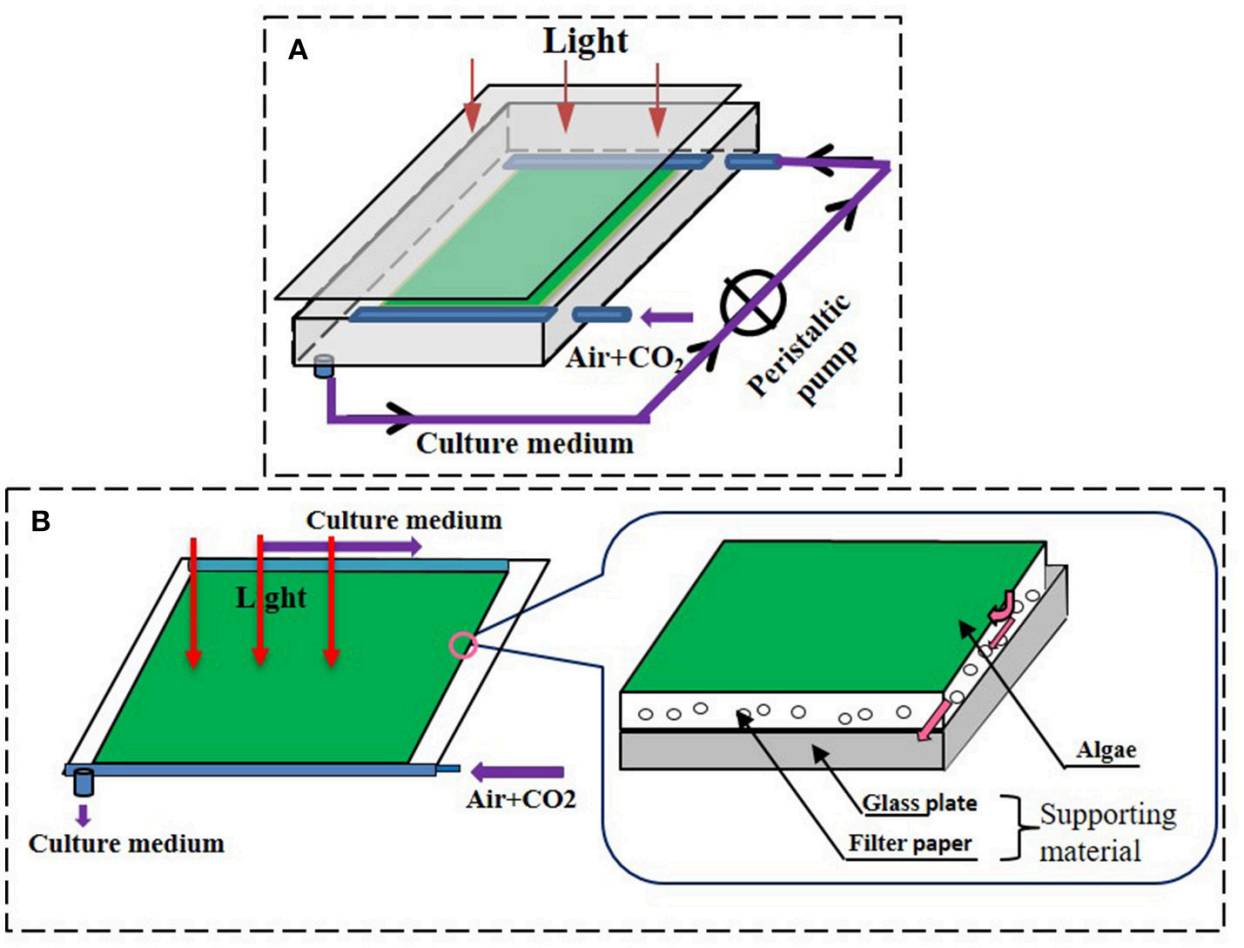
The schematic diagrams of attached cultivation devices. **(A)** Attached cultivation module of the photobioreactor, the residual medium was recycling. The medium was propelled to the system by a peristaltic pump when it flowed through the chamber during the cultivation. **(B)** The detailed structure of the cultivation surface of the attached photobioreactor. Permission to use and adapted from Cheng et al. (2014).

The biofilm attached cultivation system applied in this research was similar to the type 1 reactor used by Liu et al. (2013) and to that described by Cheng et al. (2014) (Figure 1). In brief, a glass chamber comprising a glass plate and attached algal biofilm disks was placed on an iron rack with a certain tilt angle against the horizontal plane. The medium was propelled (1.2 L in total, approximately 10 ml min^−1^) by a peristaltic pump (TP12DC 12V, Guangzhou JU PlasFitting Technology Co., Ltd., China) to facilitate circulation inside the system. Cold-white fluorescent lamps provided illumination at 100 ± 10 μmol m^−2^s^−1^ as measured inside the chamber at the position of the cultivated algae cells. Continuous air was injected into the glass chamber with a speed of 0.1 vvm and the temperature inside the glass chamber was 20 ± 2°C during the experiments. The culture time of 8 days for *C. pyrenoidosa* with biofilm attached culture was proved well in our trial test.

### Culture of *Chlorella pyrenoidosa* with swine wastewater

Swine wastewater from a private farm in Wuhan of Hubei province, China, was used as a medium to cultivate *C. pyrenoidosa*. Pre-treatment was carried out by sedimentation and filtration with a filter cloth to remove large and non-soluble particulates. After filtration, the wastewater was not autoclaved and undiluted but used directly as a medium for algal cultivation in the biofilm attached PBR. The culture conditions for control group (BG11 medium) and the raw swine wastewater were identical to those described the above section of biofilm attached cultivation system. The characteristics of the raw swine wastewater are summarized in Table 1. Remarkably, the wastewater contained heavy metals of Zn^2+^, Cu^+^, and Fe^2+^.

**Table 1:** The characteristics and features of the raw piggery wastewater.

**Parameter (mg L^−1^)**	**Fe^2+^**	**Cu^+^**	**Zn^2+^**	**TP**	**NH_4_^+^–N**	**SS**	**COD**	**BOD**
	1.8 ± 0.1	2.2 ± 0.2	2.8 ± 0.1	36.3 ± 1.2	402 ± 2.6	720 ± 4.2	601 ± 3.4	728 ± 2.2

*Data are means ± standard deviations of three replicates*.

### Analytical procedures

#### Biomass estimation

The biomass was determined with the gravimetric method (Liu et al., 2013). During the experiments, two algae disks would be collected every 2 days; cells from the filter membrane were flushed with distilled water and then filtered to a pre-weighed 0.45 μm GF/C filter membrane (Whatman, England; *DW*_0_). The membrane was oven dried at 80°C for about 24 h and then cooled down to room temperature to measure dry weight (*DW*_1_). Finally, their average was used. The *DW* was calculated as follows:

$$\begin{array}{l} \text{DW}=\left({\text{DW}}_{1}-{\text{DW}}_{0}\right)/0.001 \end{array}$$

where 0.001 represented the footprint area of the “algal disk” (m^2^).

#### Nutrients analysis in wastewater

A volume of 5 mL of recirculating medium was collected every 2 days from the attached PBRs for nutrient removal analysis. The samples were first centrifuged at 1,500 g for 10 min, and then the supernatants were filtered using a 0.45 mm nylon membrane filter. The filtrates were appropriately diluted and analyzed for COD, NH_4_^+^–N, and TP, parallel sample was made. The COD was measured using the standard potassium dichromate method based on the Chinese National Standard GB11914-1989 with a Speed Digester (Yao et al., 2015). Potentiometric analysis using a selective electrode method was used to measure ammonia (N–NH_3_) (APHA, 2012). TP were measured according to Standard Methods (Eaton et al., 2005; Hach, 2008).

#### Heavy metal elements alterations

A volume of 50 mL of culture medium was gathered, respectively, at the second day, the fourth day, the sixth day, and the last day according to the limiting of the broth, and was examined the concentration of heavy metal of copper, zinc, iron with atom absorption spectrographic methods (PinAAcle 900T, USA).

### Lipid extraction

The attached algal cells were harvested by washing down with de-ionized water and centrifugation at 3,800 g for 10 min (Allegra X-22R, Beckman coulter, USA). The algal pellets were washed three times with de-ionized water to remove any attached salt. Then the total lipid was measured according to Bligh and Dyer's method (Bligh and Dyer, 1959) and Cheng et al. (2013).

### FAMEs content analysis of algae biodiesel

FAMEs content analysis was according to Chen et al. (2012) and Wang et al. (2016). Briefly, algae powder was suspended in 2 mL 0.4 M KOH methanol solution, and was then heated at 70°C for 30 min in a water bath. After cooling, 2 mL 0.6 M H_2_SO_4_-methanol solution and 1 mL 14% BF_3_-methanol solution (Sigma-Aldrich, USA) were added, the mixture was heated in water bath at 70°C for 30 min again. After cooling, the fatty acid methyl esters (FAMEs) were extracted with 2 mL *n*-hexane, then centrifuged at 4,000 rpm for 5 min. The *n*-hexane layer was transferred to a vial. The prepared sample was (0.5 mg) was dissolved in heptane (1 mL) containing heptadecanoic acid methyl ester (C_18_H_37_COOCH_3_, 50 μg) as internal standard for FAMEs analysis on a Varian 450GC (Varian Inc., USA) equipped with a flame ionization detector (FID) and Agilent HP-5 GC Capillary Column (30 m × 0.25 mm × 0.25 μm). Nitrogen was used as carrier gas. The injector temperature was set at 280°C with an injection volume of 2 μL under split mode (10:1). The detector temperature was set at 280°C. The individual FAMEs were identified by chromatographic comparison with authentic standards (Sigma).

All experiments were performed in duplicate, and average values were reported. Results were analyzed with EXCEL (Microsoft Office Enterprise, 2010) and SPSS 11.5 for Windows (SPSS Inc., 2007); ANOVA was performed when applicable.

## Results

### Accumulation of biomass of *Chlorella pyrenoidosa* with biofilm attached cultivation

**Figure 2:**
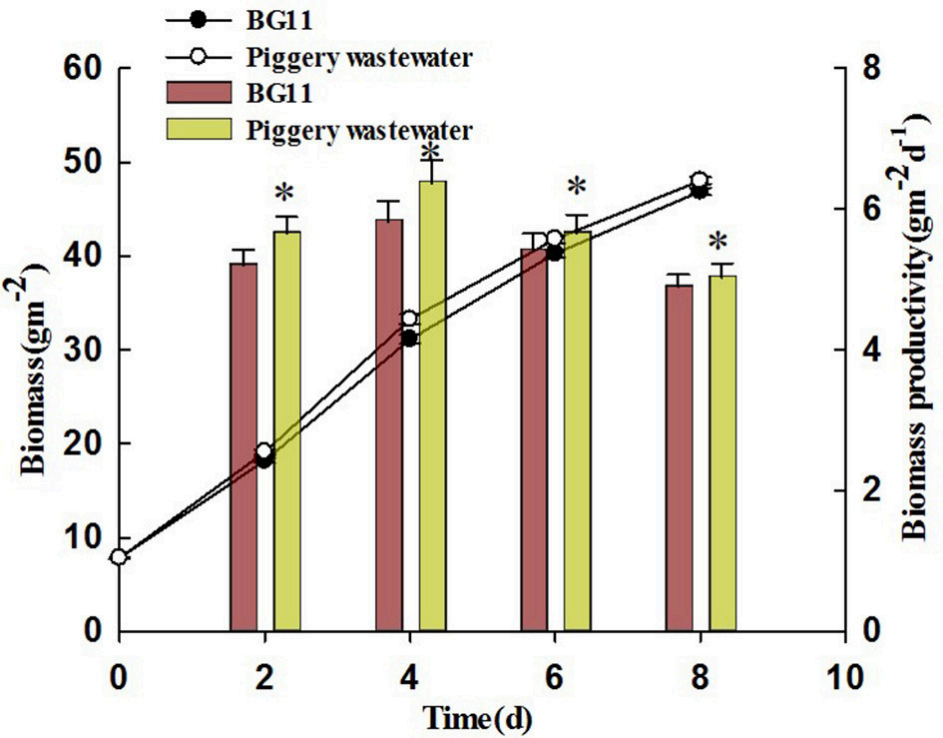
The accumulation of biomass of *Chiarella pyrenoidosa* in BG 11 (control), piggery wastewater with biofilm attached cultivation. The algal cells were cultivated in attached culture for 8 days under continuous illumination of 1 00 ± 10 μmol m^−2^ s^−1^. ^*^Indicates significant differences among treatments (*P* < 0.05), Error bars are standard deviations.

The growth curves of *C. pyrenoidosa* in swine wastewater for 8 days were showed in Figure 2. For the first 2 days, the growth was almost identical with that in BG11. After that, *C. pyrenoidosa* grew faster in swine wastewater. By the end of experiment, biomass and biomass productivity were 48.02 g m^−2^ and 5.03 g m^−2^ d^−1^, respectively, for the wastewater treatment. They were higher than that of control treatment (BG11). A smaller yet still statistically significant increase was also found under the swine wastewater for biomass productivity (repeated measures one-way ANOVA) corresponding to a mean increase of 7.3% of control.

Conventionally, in the lag state, algal cells need to adapt to the new environment since the nutrients contained in the wastewater are different from those found inside the BG11 medium. In this study, the microalgae cultivated in the biofilm attached PBR with the COD concentration of 601 mg L^−1^ had a short lag time and could quickly adapt to the environment.

### Lipid productivity and analysis of the fatty acid profile

**Figure 3:**
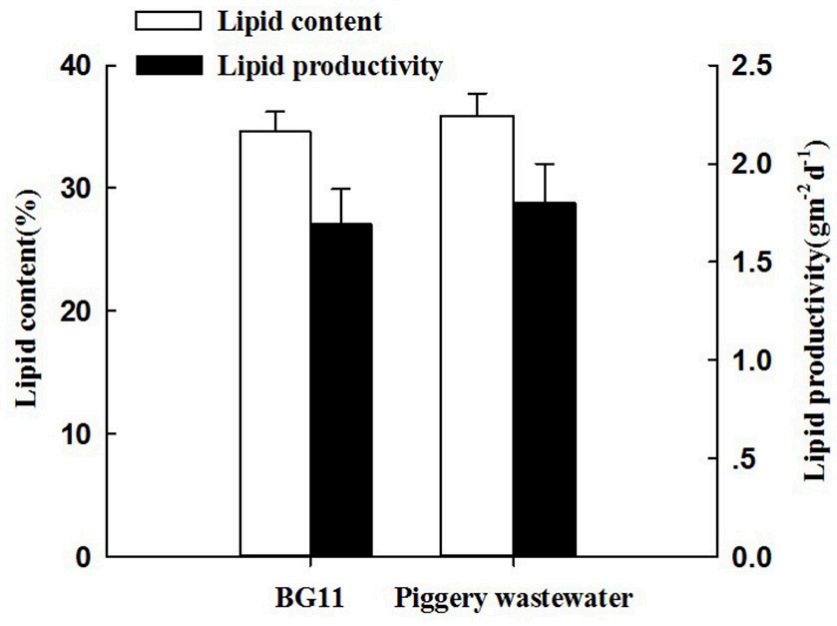
The lipid content and lipid productivity of *Chlorella pyrenoidosa* in the treatment of BG 11 (control) and piggery wastewater with biofilm attached cultivation. The algal cells were cultivated in biofihn attached culture for 8 days under continuous illumination of 100 ± 10 μmol m^−2^ s^−1^. Error bars are means ± standard deviations of three replicates.

The lipid content of *C. pyrenoidosa* in the two treatments was shown in Figure 3. The lipid content of the algae in the treatment with BG11 and swine wastewater was similar, reaching 34.6 and 35.9% and corresponded to a lipid productivity of 1.69 and 1.80 g m^−2^ d^−1^, respectively. So, there were no statistically different between the two treatments.

The compositional distribution of fatty acids extracted from the microalgae is shown in Table 2. In the present study, the FAMEs compositions of *C. pyrenoidosa* mainly consisted of C16:0 (palmitic acid methyl ester), C18:2 (octadecadienoic acid methyl ester), and C18:3 (octadecatrienoic acid methyl ester). The highest overall FAMEs yield was obtained in piggery wastewater with C18:3 (43.39% of the total fatty acids) as the most abundant fatty acid, which was higher than in BG11 medium (32.14% of the total fatty acids). Linoleic acid (C18:2) content reached 37.38% of the total fatty acids in wastewater, only 25.47% in BG11 medium. Nevertheless, the fraction of oleic acid was higher with BG11 (27.22% of the total fatty acids) than when using swine wastewater as a source of nutrients (4.43% of the total fatty acids). The fraction of palmitic acid methyl ester (C16:0) in the two treatments was similar, corresponding to 8.63% in wastewater and 9.30% in BG11. When the algae cells cultivated in BG11 media were transferred to the swine wastewater, polyunsaturated fatty acid content increased from 57.61 to 81.17%, however, the amount of monounsaturated fatty acids decreased from 27.82 to 5.08%. The unsaturated FAMEs for C16:1, C18:2, and C18:3 in biofilm attached cultures with piggery wastewater were predominant in the FAMEs profile.

**Table 2:** Effect of piggery wastewater and BG11 medium on the fatty acids composition as % of total fatty acids of *Chlorella Pyrenoidosa* with biofilm attached cultivation for 8 days.

**The content of fatty acids composition (%)**	**Piggery wastewater**	**BG11 medium**
C13:0	0.10	0.04
C14:0	0.94	0.91
C15:0	0.41	0.27
C16:0	8.63	9.30
C16:1	0.65	0.60
C17:0	0.75	0.57
C18:0	2.75	3.38
C18:1	4.43	27.22
C18:2	37.78	25.47
C20:0	0.15	0.10
C18:3	43.39	32.14
SFAs	13.73	14.57
MUFAs	5.08	27.82
PUFAs	81.17	57.61

### Nitrogen, phosphorus, and COD removal capability

**Figure 4:**
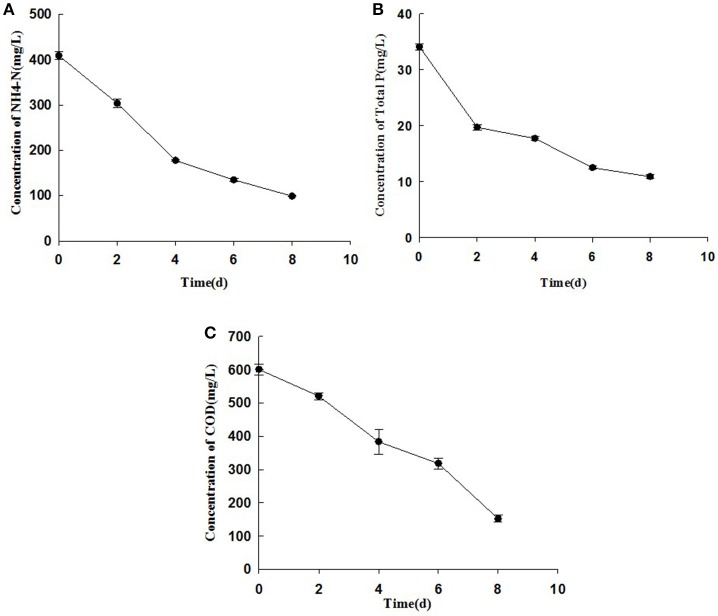
The changes of concentrations of NH^4+^–N **(A)**, TP **(B)**, and COD **(C)** of cultivation broth by *Chiarella pyrenoidosa* with biofilm attached cultivation. The algal cells were cultivated in biofilm attached culture for 8 days under continuous illumination of 100 ± 10 μmol m^−2^ s^−1^. Error bars are means ± standard deviations of three replicates.

**Figure 5:**
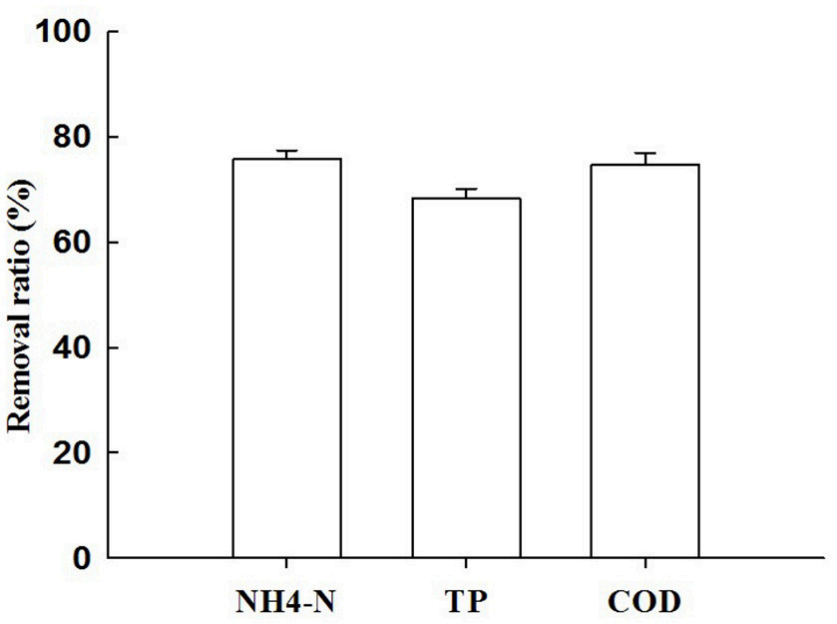
The removal ratio ofNH^4+^–N, TP, and COD with treatment ofpiggery wastewater by *Chiarella pyrenoidosa* biofilm attached cultivation. The algal cells were cultivated in biofilm attached culture for 8 days under continuous illumination of 100 ± 10 μmol m^−2^ s^−1^. Error bars are means ± standard deviations of three replicates.

Figures 4, 5 describe the removals of NH_4_^+^–N, TP, and COD achieved with treatment of piggery wastewater by microalgae cultivation. In this study, the cultivation of *C. pyrenoidosa* in the form of a biofilm receiving wastewater as a source of nutrients decreased the initial NH_4_^+^–N concentration from 409 to 98 mg L^−1^, and TP concentration from 35 to 10 mg L^−1^, and the initial COD concentration from 601 to 152 mg L^−1^. This corresponded to a removal ratio of NH_4_^+^–N, TP, and COD of 75.9, 68.4, and 74.8%, respectively.

The typical N/P ratio for optimal conditions for microalgae biomass production was 8:1 (US DOE, 2008); however, the N/P ratios in our swine wastewater were higher than that of values (Table 1). Current literature has reported that algae can assimilate NH_4_^+^–N, nitrate, and simple organic nitrogen such as, urea, acetic acid, and amino acids in wastewater; however, the concentration of NH_4_^+^–N of <300 mg L^−1^ in the accessible paper (Beuckels et al., 2015).

### Heavy metal elements disposal ratio

**Figure 6:**
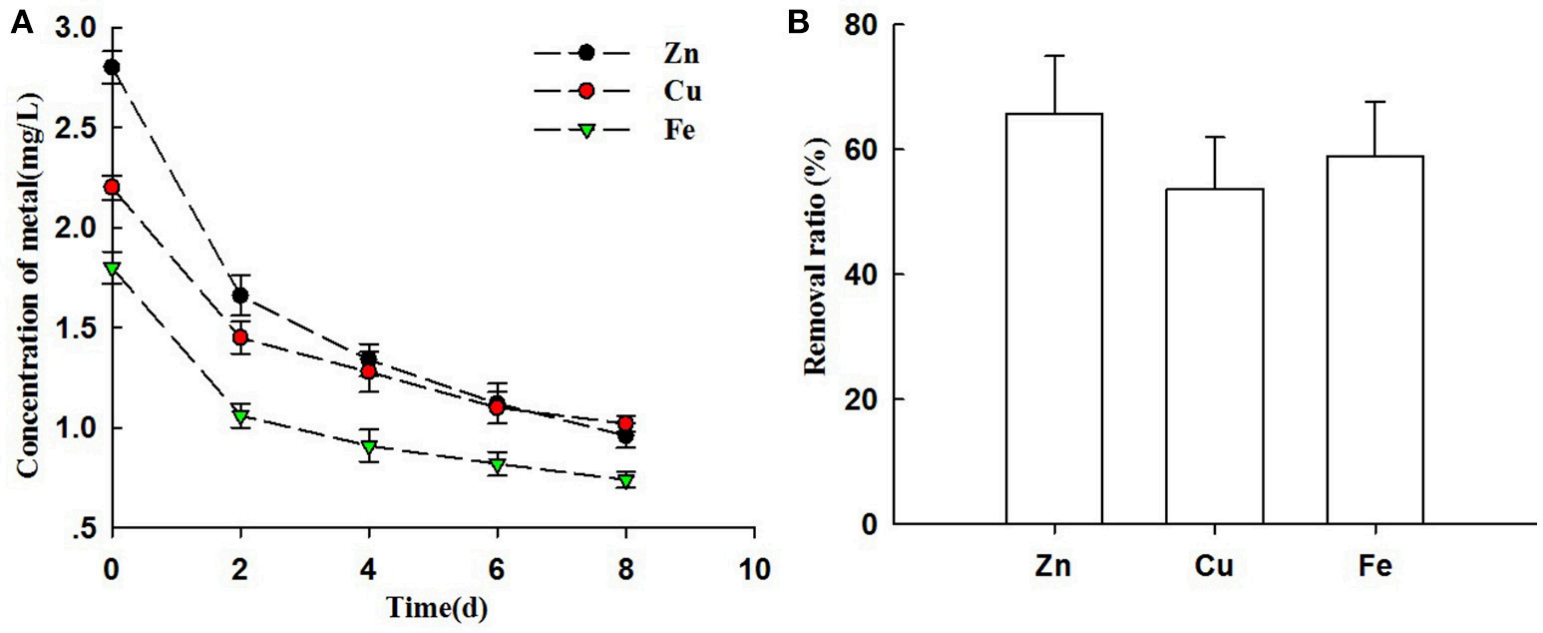
Heavy metals ions removal from piggery wastewater by biofihn attached culture (**A**) The changes of concentrations of Zn^2+^, Cu^+^, and Fe^2+^ of cultivation broth by *Chlorella pyrenoidosa* with biofilm attached cultivation. (**B**) The removal ratio of Zn^2+^, Cu^+^, and Fe^2+^ with treatment of piggery wastewater by *Chlorella pyrenoidosa* biofilm attached cultivation. The algal cells were cultivated in biofilm attached culture for 8 days under continuous illumination of 100 ± 10 μmol m^−2^ s^−1^. Error bars are means ± standard deviations of three replicates.

The swine wastewater in this study was detected some of high concentration of heavy metals ions. Table 1 showed that the concentration of Zn^2+^ in swine wastewater reached 2.8 mg L^−1^, followed by Cu^+^ and Fe^2+^ (2.2 and 1.8 mg L^−1^, respectively). Heavy metals removal from swine wastewater by microalgae with attached culture has been tested (Figure 6). As shown in Figure 6A, the metal concentrations (Zn^2+^, Cu^+^, and Fe^2+^) decreased sharply after only 2 days of cultivation. The reduction rate greatly decreased in the following days. By the end of experiment, residual quantity of Zn^2+^, Cu^+^ and Fe^2+^ were still detected reaching concentrations of 0.96, 1.02, and 0.74 mg L^−1^, respectively (Figure 6A).

This result indicated the high removal ratio of Zn^2+^ (65.71%), Cu^+^ (53.64%), and Fe^2+^ (58.89%) with the attached cultivation of *Chlorella* (Figure 6B). Our results are supported from Carrilho and Gilbert (2000) who revealed that biosorption of algae was rapid and accumulation of metals happened within short time (Figure 6A).

## Discussion

Ayre et al. (2017) reported that many microalgae species can grow in diluted digested piggery wastewater, and they utilize the abundant N, P, and other organic carbon in the water. *Chlorella* sp. was the dominant algae species in a wastewater treatment study. However, nearly all experiments were performed on piggery wastewater diluted with either synthetic media or water to reduce the toxicity of organic components (Min et al., 2011; Zhu et al., 2011). Min et al. (2011) and Zhu et al. (2011) also cultivated *Chlorella* sp. in swine wastewater with relatively high COD in a pilot-scale PBR, however, in their experiment the biomass productivity and initial NH_4_^+^–N concentration of wastewater were lower than our experiment (Figure 2). Flynn (1991) reported that ammonium assimilation causes a rapid and reversible inactivation of nitrate transport which influences growth of microalgae.

The dilution of swine wastewater for microalgae growth would be limited by the high cost of clean water consumption, sewage volume, need for artificial nutrients, and other inconvenient operations. Therefore, developing an efficient process that allow algae to grow well under undiluted piggery slurry is very important, rendering the application of microalgae-based technology feasible and economical for full-scale wastewater treatment and biodiesel production. This study aimed at investigating the feasibility of tested microalgae in undiluted slurry of piggery wastewater, and the microalgae treated with the biofilm attached culture method grew well in undiluted slurry.

Feng et al. (2012) and Rodolfi et al. (2009) proved that lipid accumulation can be raised through increasing light intensity. The wastewater had a darker color than the BG11; however, lipid accumulation for the swine wastewater treatment was not inhibited in biofilm attached cultivation. This might be because the algal cells cultivated with attached PBR are generally immobilized and fixed onto supporting materials in high density and separated with swine wastewater. *Chlorella* sp. are known to have high content of lipids, which have the potential to be converted to biofuels that can be used mixed to—or in substitution to traditional fossil fuels. Beal et al. (2012) reported the cost of algal biofuel production was mainly related to algal culture (77%), harvesting (12%), and lipid extraction phases (7.9%). It was obvious that the utilization of wastewater as a substitute for algae nutrients would significantly reduce the operational cost of algae cultivation (Cai et al., 2013).

The environmental and cultural conditions have been reported by previous research to have an effect on lipid content and fatty acid (Petkov and Garcia, 2007; Li et al., 2011). So, it was not surprising to observe the different FAMEs profiles in our study (Table 2). Not all lipids can be converted to FAMEs, such as, glycolipids and phospholipids and long-chain carbon (Li et al., 2011). However, *C. pyrenoidosa* cultivated in swine wastewater was found to have shorter carbon chains for fatty acids in our study. The degree of unsaturation of fatty acids is due to the metals toxicity more than to favoritism of piggery wastewater. They mainly contained 16–18 carbons (Table 2), which were ideal for biodiesel conversion (Huang et al., 2010). Integration of microalgal cultivation with wastewater for biodiesel production is a promising choice and could enhance the economic and environmental sustainability (Lee and Lee, 2002).

The high NH_4_^+^–N content in wastewater represents a growth inhibitory factor for microalgae because it may contribute to changes in pH value of the culture medium. Tam and Wong (1996) showed that the growth of *Chlorella* sp. was reduced at NH_4_^+^–N concentrations higher than 700 mg L^−1^ with a pH level below 7. Chiu et al. (2015) reported that the TP concentration for biomass productivity was lower than 100 mg L^−1^. The consumed phosphorus of swine wastewater was mainly assimilated by algal cells. The effects of NH_4_^+^–N and TP concentrations in wastewater on the *Chlorella* biomass production and productivity are similar (Chiu et al., 2015). The consumed COD in this study with attached cultivation of *Chlorella* were higher than the reported that of 73.18% COD removal ratio with *Arthrospira (Spirulina) platensis* cultivated in 25% olive oil mill wastewater (Markou et al., 2012).

The main physicochemical approaches to remove heavy metal ions from wastewaters include ion exchange, chemical precipitation, electrokinetic, membrane processing, and adsorption (Lee and Pandey, 2012; Goharshadi and Moghaddam, 2015). The incomplete removal of heavy metal ions and high costs of chemicals are the main limiting factors in the development of physicochemical approaches. However, biosorption of heavy metal ions in wastewater with proper microalgae species could offer an ecologically safer, cheaper, and more efficient means. Indeed, algae could be used to absorb toxic and radioactive metal ions and to recover precious metals (Pohl and Schimmack, 2006). And polysaccharides and proteins present in algae cell walls contain the most number of metal binding sites (Schiewer and Volesky, 2000). However, factors including concentration of metal ions, algae biomass, pH, temperature, and presence of competing ions affect the biosorption of heavy metals ions by algae. Algal cell walls are the first barrier against biosorption of heavy metal ions. Given the different distribution and abundance of cell wall compositions in different algal strains, the biosorption capacity for metal ions would vary depend on the considered algal strain.

*Chlorella* can effectively grow and utilize nutrients in swine wastewater, reducing the content of metal ions in this research. Table 3 compared of the growth, lipid production, and cultivation parameters of microalgae in wastewater for different cultivation methods. It could be observed that most of study concerned of algae-based treatment diluted wastewater (Ji et al., 2013), which would increase cost in practice. In addition, few papers focused on the question of heavy metal pollution of piggery wastewater, which recently has attracted growing attention to agricultural pollution (Zeraatkar et al., 2016). The cultivation scale of most recent studies was limited to the laboratory bench—or pilot plant scale. The significant question to consider is what kind of cultivation system could be more economically viable and environmental-friendly for large scale production of microalgal biomass. As a result of the difficulty of construction and the condition of vast land area, most of the PBRs and ponds used in recent studies are not economically convenient for large-scale microalgal biomass production. Therefore, the efforts on novel cultivation system are very important in the future research (Wang et al., 2015).

**Table 3:** Comparison of growth, lipid production and cultivation parameters of microalgae in wastewater for different cultivation methods.

**Wastewater source**	**Algae**	**Biomass**	**Lipid content (%)**	**NH_4_–N (mg L^−1^)**	**TP (mg L^−1^)**	**COD (mg L^−1^)**	**Cultivation systems**	**Reference**
Piggery wastewater	*Chlorella Pyrenoidosa*	48.02 g m^−2^	35.9	409	35	601	Attached photobioreactor	This study
50% Piggery wastewater	*Chlorella zofingiensis*	2.01 g L^−1^	34.8	–	146	–	Tubular bubble column photobioreactors	Zhu et al., 2011
Municipal wastewater	*Scenedesmus* sp.	0.78 g L^−1^	12.7	27.7	7.3	273.5	Erlenmeyer flasks	Sacristán de Alva et al., 2013
10% Dairy wastewater	*Chlorella zofingiensis* G1	0.14 g L^−1^	17.9	5	15	–	ponds	Huo et al., 2012
Digested dairy manure	*Chlorella* sp.	1.39 g L^−1^	10.1	100	18	–	Erlenmeyer flasks	Wang et al., 2010

*“–” Means was not studied*.

In summary, *C. pyrenoidosa* could accumulate considerable biomass and lipid in swine wastewater with biofilm attached culture. A major portion of FAMEs compositions were ideal for biodiesel conversion. The removal of NH4^+^–N, TP, COD and, as well as heavy metal pollution of wastewater with *C. pyrenoidosa* by biofilm attached culture can be a remarkable solution for swine wastewater resource utilization.

## Author contributions

PC and DL proposed the idea and hypothesis. PC drafted the manuscript. YW carried out the experiment design and carried out the biofilm attached cultivation of *C. pyrenoidosa* in undiluted swine wastewater. TL performed the statistical analysis and helped to draft and revise the manuscript. All authors read and approved the final manuscript for publication.

### Conflict of interest statement

The authors declare that the research was conducted in the absence of any commercial or financial relationships that could be construed as a potential conflict of interest.
